# Correlation of MET-Receptor Overexpression with MET Gene Amplification and Patient Outcome in Malignant Mesothelioma

**DOI:** 10.3390/ijms222312868

**Published:** 2021-11-28

**Authors:** Eric Santoni-Rugiu, Maya Jeje Schuang Lü, Jan Nyrop Jakobsen, Linea Cecilie Melchior, Jesper Ravn, Jens Benn Sørensen

**Affiliations:** 1 Department of Pathology/Danish National Mesothelioma Center, Rigshospitalet, Copenhagen University Hospital, DK-2100 Copenhagen, Denmark; linea.cecilie.melchior@regionh.dk; 2 Biotech Research & Innovation Centre (BRIC), University of Copenhagen, DK-2200 Copenhagen, Denmark; 3 Department of Oncology/Danish National Mesothelioma Center, Rigshospitalet, Copenhagen University Hospital, DK-2100 Copenhagen, Denmark; maya.jeje.schuang.lue@regionh.dk (M.J.S.L.); jannyrophan@yahoo.com (J.N.J.); 4 Department of Thoracic Surgery/Danish National Mesothelioma Center, Copenhagen University Hospital, DK-2100 Copenhagen, Denmark; jesper.ravn@regionh.dk

**Keywords:** MET, overexpression, gene amplification, malignant mesothelioma, immunohistochemistry, fluorescence in situ hybridization, patient outcome

## Abstract

Thanks to clinically newly introduced inhibitors of the mesenchymal–epithelial transition (MET) receptor tyrosine-kinase, MET-gene copy number gain/amplification (MET-GCNG/GA) and increased expression of the MET protein are considered very promising therapeutic targets in lung cancer and other malignancies. However, to which extent these MET alterations occur in malignant mesothelioma (MM) remains unclear. Thus, we investigated by well-established immunohistochemistry and fluorescence in situ hybridization methods, the frequency of these alterations in specimens from 155 consecutive MMs of different subtypes obtained from pleural or peritoneal biopsies and pleurectomies. Thirty-three benign reactive mesothelial proliferations (RMPs) were used as controls. MET-protein upregulation was observed in 35% of all MM-cases, though restricted to predominantly epithelioid MMs. We detected low-/intermediate-level MET-GCNG/GA in 22.2% of MET-overexpressing MMs (7.8% of whole MM-cohort) and no MET-GCNG/GA in the other 77.8%, suggesting other upregulating mechanisms. In contrast, 100% of RMPs exhibited no MET-upregulation or MET-GCNG/-GA. Neither MET exon 14 skipping mutations nor MET-fusions were detected as mechanisms of MET overexpression in MM using RNA next-generation sequencing. Finally, in two cohorts of 30 MM patients with or without MET overexpression (MET-positive/-negative) that were matched for several variables and received the same standard chemotherapy, the MET-positive cases showed a significantly lower response rate, but no significant difference in progression-free or overall survival. Our results imply that MET overexpression occurs in a substantial fraction of predominantly epithelioid MMs, but correlates poorly with MET-amplification status, and may impact the likelihood of response to mesothelioma standard chemotherapy. The predictive significance of MET-IHC and -FISH for possible MET-targeted therapy of MM remains to be elucidated.

## 1. Introduction

The transmembrane mesenchymal–epithelial transition (MET) receptor tyrosine-kinase (RTK) is the receptor for hepatocyte growth factor (HGF), is encoded by the MET proto-oncogene located on chromosome 7q21-31, and is normally expressed in several epithelial and mesenchymal cell types. MET can activate several signaling pathways, including the PI-3K/AKT, RAS-Rac/Rho, RAS-MAPK, JAK-STAT3, and phospholipase C pathways [1,2]. Thereby, MET regulates cell proliferation, survival, differentiation, and motility during processes such as morphogenesis, angiogenesis, cell proliferation, migration, invasiveness, and metastasis development [1,2]. MET signaling has been found to be deregulated in a variety of cancers through different mechanisms, such as overexpression of HGF or MET protein and activating point mutations in the TK domain or affecting the splicing site donor and acceptor regions near exon 14 of the MET gene (so-called “exon 14 skipping mutations”). The latter results in alternative splicing, skipping of exon 14 and inhibition of MET-protein degradation, ultimately leading to accumulation of catalytically active MET. Additional mechanisms of constitutive activation of MET signaling in cancer cells are MET gene fusions and MET-gene copy number gain/amplification (MET-GCNG/GA) [1,2]. Consequently, dysregulated MET represents an attractive potential therapeutic target in several cancers.

The best example until now has been non-small-cell lung cancer (NSCLC), in which MET exon 14 skipping mutations affecting the corresponding splicing sites and MET-GCNG/GA occur in 3–5% and 1–6% of cases, respectively [2,3,4,5]. Overexpression of MET protein has been reported in 25 to 75% of cases, depending on the antibody and cutoff value used in the implemented immunohistochemistry (IHC) assays as well as the histological NSCLC subtypes analyzed [2,3,4,5]. MET overexpression, MET-GCNG/GA, and MET exon 14 skipping mutations are considered negative prognostic markers in NSCLC [3,4,5]. Inhibition of MET signaling is currently achievable in NSCLC patients using the MET/ALK/ROS tyrosine-kinase inhibitor (TKI) Crizotinib or selective MET-TKIs (Capmatinib, Savolitinib, Tepotinib, Cabozantinib), or MET or HGF monoclonal antibodies (mAbs), as well as MET or HGF antibody–drug conjugates [2,3,4,5]. In addition to MET exon 14 skipping mutations identified by next-generation sequencing (NGS) of DNA or RNA, MET-expression analyzed by IHC and MET-GCNG/GA status evaluated by fluorescence in situ hybridization (FISH) are considered biomarkers for targeted anti-MET therapy in NSCLC [3,4,5]. Given intratumor heterogeneity, direct morphological assessment of MET-overexpression and MET-GCNG/GA in tumor specimens using IHC and FISH is deemed to be more sensitive than NGS-based assessment of MET-amplification and more informative as to whether the deregulated MET-receptor signaling can be targeted in a specific patient [4]. For this reason, FISH has been the first approved and effective method employed to select NSCLC patients with MET-amplification in clinical trials with MET inhibitors [2,3,4,5].

Malignant mesothelioma (MM) is a very aggressive and challenging cancer type originating from mesothelial cells coating the pleura (roughly 90% of all MMs), or more rarely other serosal membranes, such as the peritoneum (approximately 10% of all MMs), pericardium or tunica vaginalis testis (<1%) [6]. Its pathogenesis is associated with exposure to asbestos or asbestos-like fibers and inactivation of tumor suppressor genes such as BAP1, CDKN2A, NF2, TP53, SETD2, or LATS2 [6,7,8,9] as main cancer drivers, which represents a significant challenge for effective targeted therapy of MM. Given that for the last two decades the approved frontline treatment for MM has been chemotherapy with the combination of platin and pemetrexed, to which MM is very often poorly responsive, there is a strong unmet need for more specific therapeutic targeting of this malignancy based on its molecular alterations. In this respect, functional genomic studies have revealed that oncogenic gain-of-function alterations are rare in MM, but have shown nonetheless the involvement of deregulated signaling pathways and cellular processes, including those depending on the activation of transmembrane RTKs, in the pathogenesis of MM (reviewed in [9]). The possible reliance of MM cells on these alterations could represent vulnerabilities to be exploited as potential therapeutic targets and is worth being investigated in detail.

With regard to RTKs, how MET is expressed in MM and whether it could represent a target for this cancer type remains to be further elucidated. Although, preclinical investigations indicate that the HGF-MET axis may play an important role in mesothelioma-genesis [10] and that MET can be upregulated in MM cell lines [11], MET-mutations are extremely uncommon in MM tissue specimens or MM cell lines [7,8,9,11]. Similarly, these genomic analyses have not reported MET-GCNG/GA in pleural MM [7,8,9], though one study described 1 out of 13 genomically profiled peritoneal MMs with high-level MET-amplification, suggesting that a minor subset of these tumors could be driven by this alteration [12]. Moreover, it is unclear whether the apparently low detection rate of MET-GCNG/GA in MM using DNA/RNA-sequencing techniques could in fact reflect intratumor heterogeneity resulting in the presence of this MET alteration only in minor, difficult-to-detect MM cell populations within the tumor tissue. In any case, overexpression of the MET receptor or its ligand HGF have been described in some cases of MM and in certain MM cell lines displaying functional activity of MET signaling, whereas the normal mesothelium has been reported devoid of MET expression [9,11]. Collectively, these data suggest that a subgroup of MMs may depend on the expression and activation of the MET receptor. Yet, only a few studies exploring the incidence of MET-GCNG/GA by FISH and/or increased protein expression using IHC in MM have been reported. These investigations have shown variable results attributable to differences in types of specimens, specificity/sensitivity of analytical methods, and scoring procedures [13,14,15]. Thus, to further clarify how MET is expressed in MM, we investigated the frequency of MET-GCNG/GA and expression by FISH and IHC in 155 consecutive MM cases, employing strictly defined criteria previously applied to a cancer type with significant heterogeneity of MET abnormalities, such as NSCLC [16,17]. Furthermore, we addressed using RNA next-generation sequencing (RNA-NGS) whether MET overexpression in MM samples that did not display corresponding MET-GCNG/GA could be caused by MET exon 14 skipping mutations. Finally, we examined whether MET aberrations had a prognostic impact or predicted likelihood of response to chemotherapy by comparing two groups of MM patients with and without MET overexpression treated at our institution during 2015–2017, i.e., with a minimum 3 years of follow-up.

## 2. Results

### 2.1. MET Gene Transcript Profile in MM

**Figure 1:**
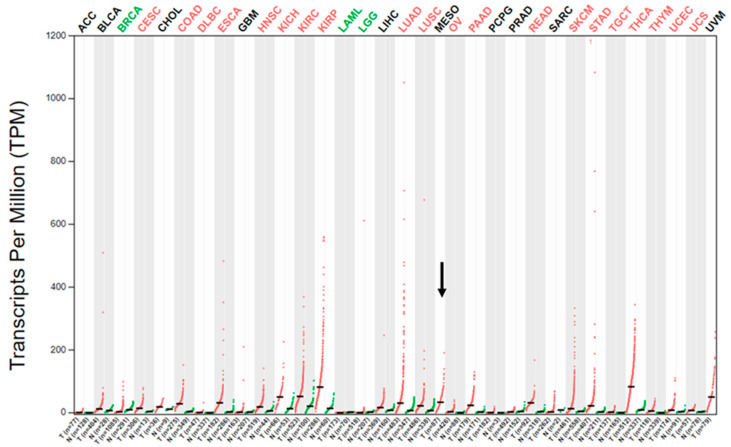
MET gene expression profile in different cancer types as part of the TCGA. Dot plot of the MET transcript expression profile across the different TCGA tumor samples (red dots) and paired normal tissues (green dots) adapted from the public GEPIA web server [18,19]. The horizontal black bars represent median values. The abbreviations for the analyzed cancer types are explained in Table S1. MESO is the abbreviation for MM, which is represented without paired normal tissue and is further indicated by the black arrow.

As initial assessment of MET gene expression in human MM, we looked at the expression of the MET mRNA in the public GEPIA database for gene expression profiling analysis in the different cancer types sampled for “The Cancer Genome Atlas” (TCGA) project [18,19]. The database contains data from 87 MMs collected as part of the TCGA, as initially described by Hmeljak et al. [8]. The GEPIA’s dot plot of the MET transcript expression profile across the different TCGA tumor samples (and for most tumors, but not MM, also paired normal tissues) (Figure 1) showed a median expression level in MM comparable to other cancer types, such as lung squamous carcinoma and adenocarcinoma, colorectal adenocarcinomas, pancreatic adenocarcinoma, or esophageal and gastric carcinoma, though the expression range was much wider in these malignancies than in MM, particularly in lung adenocarcinoma, which could reflect the fact that a subset of this cancer type is characterized by high-level MET-amplification [4,5,16,17]. Moreover, the median expression level of MET transcript in MM was higher than that in mammary, ovarian, and prostatic adenocarcinomas or glioblastomas, but lower than in melanomas, thyroid carcinomas or papillary renal cell carcinoma. Although it is difficult to extrapolate data regarding the actual MET-receptor expression in MM from this analysis, the data from GEPIA indicated that the MET gene transcript is expressed at significant levels in MM. Thus, we decided to use IHC to further elucidate the expression of this receptor in human MM.

### 2.2. Expression of MET-Receptor in MM Assessed by IHC

For this study, we immunostained formalin-fixed, paraffin-embedded (FFPE) tissue sections according to a previously established protocol utilizing the anti-MET SP44 monoclonal antibody and a MET-expression scoring system [17]. As described in Materials and Methods, endothelial cells or bronchial/alveolar epithelial cells were used as internal positive control (Supplementary Figure S1). Before analyzing the main cohort of MM cases for the study, we assessed whether the neoadjuvant platin-pemetrexed chemotherapy provided to operable MM patients would have any effect on MET expression in their pleurectomy/decortication (P/D) specimens as compared to their chemotherapy-naïve diagnostic biopsies. Thus, we performed a pilot immunohistochemical investigation on archival MM samples that we had used in previous reports [20,21,22] and, among them, we identified 10 diagnostic biopsies displaying “MET-negative” expression (immunoscore 0/1+ as defined in the Materials and Methods), 10 displaying a MET immunoscore of 2+, and 10 displaying an immunoscore of 3+. Thereafter, we compared these results with the immunostainings in their corresponding patient-matched P/D specimens. We detected no difference in the paired samples, i.e., all originally MET-negative cases continued to display an immunoscore of 0/1+ in their corresponding P/D specimens, and by the same token, all the cases with diagnostic biopsies displaying a MET immunoscore of 2+ and 3+ maintained the same expression in the tissue sections from the patient-matched P/D samples (a representative example is shown in Supplementary Figure S2).

**Figure 2:**
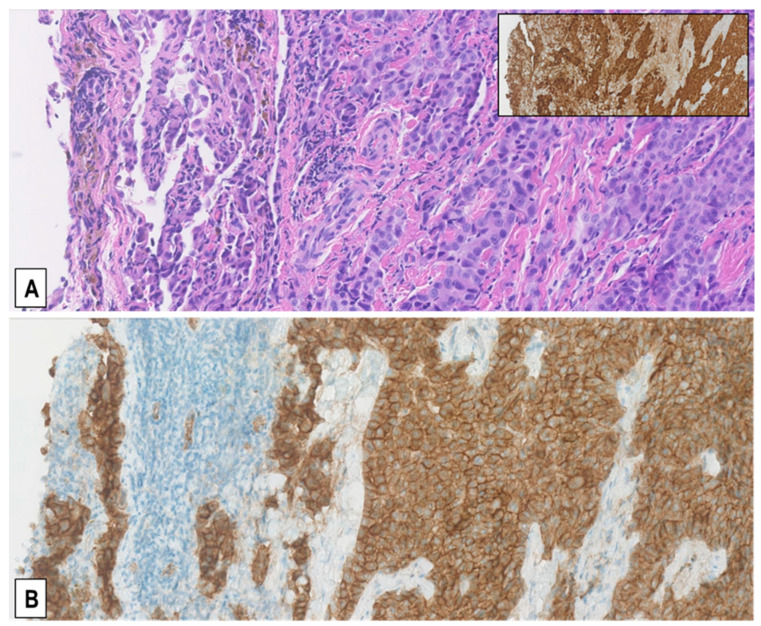
Predominantly membranous pattern of MET expression in MMs. Representative serial sections from a resected pleural epithelioid MM stained with H&E (**A**) and immunostained with anti-MET SP44 mAb (**B**) showing moderately upregulated MET expression (MET immunoscore 2+), which is more intense on the cell membrane than in the cytoplasm. Immunostaining of the same area for the mesothelial marker calretinin is shown in the inset. (Magnification (**A**,**B**), ×200).

Consistent with the fact that MET is a transmembrane RTK, we also observed in both diagnostic biopsies and P/D specimens that the expression of MET was predominantly in the membrane of tumor cells, though a certain positivity was also visible to a lesser extent in the cytoplasm (Figure 2). Hence, we concluded from the pilot study that neoadjuvant chemotherapy caused no significant change in the expression of MET in the P/D tissue samples as compared to patient-matched diagnostic biopsies and that, in our main study, we could correlate the results obtained in diagnostic biopsies with those in P/D specimens.

Accordingly, we examined a cohort of 155 unselected consecutive MM cases treated at our institution between 2015 and 2017. The demographic and pathological characteristics of the MM cases and of control cases with non-neoplastic reactive mesothelial proliferation (RMP) are described in Table 1 and the Materials and Methods.

**Figure 3:**
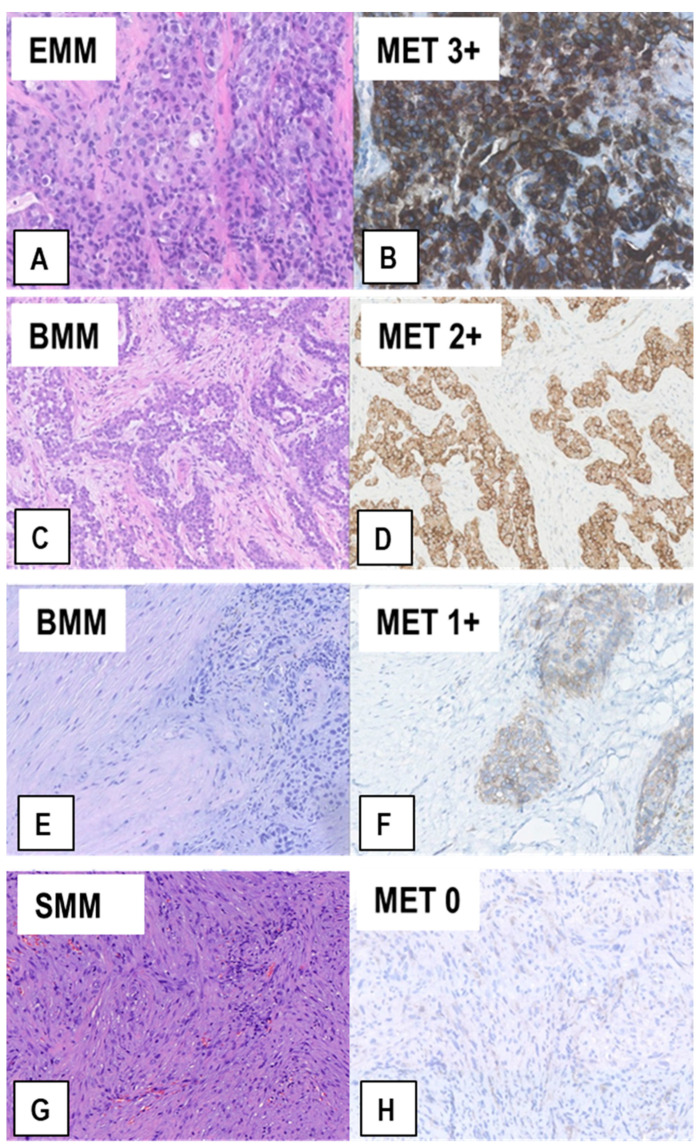
Representative examples of MET expression in tissue sections from MM of different histological types. Serial sections from each case were stained with H&E (**A**,**C**,**E**,**G**) and immunostained with anti-MET SP44 mAb (**B**,**D**,**F**,**H**). In (**A**,**B**), an EMM showing strong upregulation of MET expression (MET immunoscore 3+). In (**C**,**D**), a BMM with moderately upregulated MET expression (MET immunoscore 2+), which is only present in the epithelioid component. In (**E**,**F**), a BMM with weak MET expression (MET immunoscore 1+), which is only present in the epithelioid component, whereas the sarcomatoid component is negative. In (**G**,**H**), a SMM with no MET expression (MET immunoscore 0), in which scattered reactive endothelial cells with quite weak MET expression can be observed. (Magnification: (**A**,**B**), ×320; (**C**–**H**) ×200).

The samples from this patient cohort included FFPE tissue sections from 110 diagnostic biopsies of treatment-naïve MM and from 45 extended P/Ds performed after 3 cycles of neoadjuvant cisplatin-pemetrexed. After the immunostainings, we observed that 27 (17.4%) of the 155 MM specimens exhibited a MET immunoscore of 3+ (representative example in Figure 3A,B), 27 (17.4%) a MET score of 2+ (Figure 3C,D), and the remaining 101 (65.2%) a MET score of 1+ (65/155, 41.9%; Figure 3E,F) or 0 (36/155, 23.2%; Figure 3G,H). Twenty-three of the 27 (85%) MMs with an immunoscore of 3+ were epithelioid MMs (EMMs) (Figure 3A,B), whereas the other 4 (15%) were biphasic MMs (BMMs) (*p* < 0.05, *t*-test). Similarly, of the 27 MMs with an immunoscore of 2+, 16 (59.3%) were EMMs and 11 (40.7%) BMMs (*p* < 0.05, *t*-test). Interestingly, in the 15 BMMs with a score of 2+/3+, only the epithelioid component accounted for that, while sarcomatoid cells consistently exhibited a score of 1+/0 (example in Figure 3C,D). Accordingly, none of the 10 analyzed sarcomatoid MMs (SMMs) displayed a MET immunoscore of 3+ or 2+, but only 1+ (n = 3) or 0 (n = 7) (example in Figure 3G,H).

**Figure 4:**
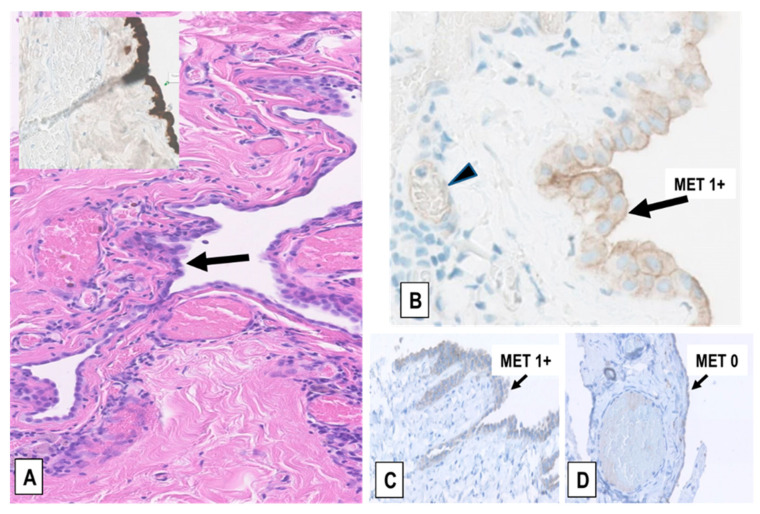
Examples of MET expression in RMPs. (**A**) RMP in a pericardial cyst (H&E staining). The hyperplastic mesothelium covering the cyst wall is indicated (arrow) and highlighted by immunostaining for the mesothelial marker calretinin (inset). (**B**) Detail of the weak membranous MET expression (immunoscore 1+) in the hyperplastic mesothelium of the pericardial cyst represented in (**A**) (arrow) and in the endothelial cells of capillaries (arrowhead), but not in leukocytes. (**C**,**D**) Two representative cases of pneumothorax-induced pleural RMP and fibrosis showing weak (immunoscore 1+) and no MET expression (immunoscore 0) in the mesothelium, respectively (arrows), and no expression in the underlying fibrotic tissue, which in (**D**) contains dilated capillaries that are negative too, in this case. (Magnification in (**A**), inset, (**C**), and (**D**): ×200; in (**B**): ×400).

For comparison to non-malignant mesothelial proliferations, 33 FFPE tissue sections from RMPs identified in FFPE surgical samples of patients operated for non-malignant pulmonary/mediastinal diseases were immunostained for MET expression with the same protocol used for MM samples. These samples exhibited variable pleural or pericardial chronic inflammation associated with mesothelial hyperplasia and various degrees of fibrosis, thus representing non-neoplastic controls for the three types of MM. Ten of the 33 (30.3%) RMPs showed a MET immunoscore of 1+ (example in Figure 4A–C) and the remaining 23 RMPs (69.7%) an immunoscore of 0 (Figure 4D), while none of the RMPs displayed upregulation of MET corresponding to a score of 2+/3+. Collectively, the results indicated that overexpression of MET (immunoscore 2+/3+) is related to malignant mesothelial proliferation, is significantly more common in EMM than in BMM or SMM and is only present in the epithelioid component of BMMs.

### 2.3. Assessment of MET-GCNG/GA in MM by FISH

**Figure 5:**
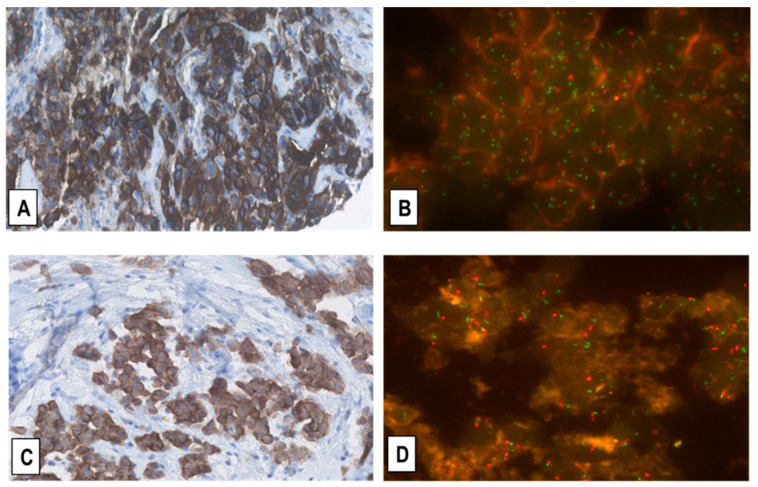
Representative immunostainings for MET-receptor expression (**A**,**C**) and corresponding FISH for MET-GCN (**B**,**D**) in two EMMs. (**A**,**B**) shows the same EMM case as in Figure 3A,B with MET overexpression (immunoscore 3+) and intermediate-level MET-GCNG/GA, as defined in the Materials and Methods. (**C**,**D**) shows another EMM with moderate upregulation of MET expression (immunoscore 2+) and no MET-GCNG/GA. (Magnification (**A**,**C**) ×200; (**B**,**D**) ×1000; In (**B**,**D**) MET signals = green; CEN7 signals = red).

The FISH analysis in the 155 MM samples showed that the average MET-gene copy number (MET-GCN)/tumor cell varied between 1.44 and 5.69. By matching the FISH results with the corresponding IHC data, we observed that MM samples displaying no upregulation of MET expression (immunoscore 0/1+) had a lower average MET-GCN/cell (between 1.44 and 2.99) than MMs with upregulated MET-receptor expression (immunoscore 2+/3+; GCN/cell = 1.54–5.69). Although significant (*p* < 0.05), this difference suggests that MMs harbor fairly modest increases of MET-GCN as compared to other cancer types such as NSCLC, in which de novo high-level MET-amplification, often with more than 15 MET gene copies (“gene clusters”), can be detected by FISH analysis [16,17]. In addition, we identified only 3 out of the 155 (2%) MM cases with intermediate-level MET-GCNG/GA; all three were pleural EMMs exhibiting a MET immunoscore of 3+ by IHC (Figure 5). We also detected 9 (6%) cases with low-level MET-GCNG/GA, of which 6 were EMMs (5 pleural and 1 peritoneal) and 3 BMMs (all pleural) showing a MET immunoscore of 3+ (6 EMMs, 1 BMM) or 2+ (2 BMMs) by IHC. Furthermore, the MET/CEN7 ratio in the MM cohort varied from 0.78 to 1.94, with the cases with ratio <1 explainable by the occurrence of amplicons including CEN7 but without a numeric balanced MET-GCNG, as observed in certain cases of NSCLC [16,17].

Together, the data implied that none of the 155 cases harbored high-level MET-GCNG/GA according to the definition criteria described in the Materials and Methods (i.e., MET/CEN7 ratio ≥2.0 or an average MET-GCN/cell ≥6.0 or ≥10% of tumor cells with “clusters” of ≥15 MET signals). However, one of the cases eventually classified with intermediate-level MET-GCNG/GA had a borderline value of 5.69 for average MET-GCN/cell and a borderline MET/CEN7 ratio of 1.94. For each sample, we analyzed 100 tumor cell nuclei, taking into account possible zonal heterogeneity of GCN, i.e., we assessed 20 neighboring tumor cell nuclei from five random areas of homogenous distribution of MET signals, as previously described [16,17]. Nevertheless, we did not observe any obvious variation in GCN from area to area of each sample, suggesting that MET-GCNG tend to be homogeneous in MM, as opposed to NSCLC [16,17]. Thus, it is unlikely that the abovementioned borderline values for MET-GCN/cell and MET/CEN7 ratio in the case classified with intermediate-level MET-GCNG/GA were due to heterogeneity and could have reached values for high-level GA in other areas. To further exclude that, we analyzed an extra population of 100 tumor cell nuclei from five additional random areas in that sample and obtained very similar borderline values of MET-GCN/cell and MET/CE7 ratio (5.65 and 1.91, respectively).

Finally, no cases with low- or intermediate-level MET-GCNG/GA showed a MET immunoscore of 1+ or 0 by IHC. Accordingly, all 10 SMMs and all 33 cases of RMPs, which as mentioned above, all exhibited a MET immunoscore of 1+/0 by IHC, showed no MET-GCNG/GA.

### 2.4. MET Overexpression in MM Specimens Is Not Associated with MET Exon 14 Skipping Mutations

Given that, in our cohort of MM specimens, the IHC and FISH analyses displayed only a limited correlation between MET overexpression and MET-GCNG/GA, we addressed the question, whether in the specimens displaying MET overexpression without concomitant MET-GCNG/GA, the receptor overexpression could be explained by alternative alterations of the MET gene. Indeed, in NSCLC, another mechanism of MET overexpression is represented by mutations involving the juxtamembrane domain of the MET gene. These mutations cause aberrant splicing of the MET transcripts, in which exon 14 is skipped, resulting in reduced degradation of the MET receptor that consequently becomes overexpressed and can function as an oncogenic driver [3,4,5]. Thus, we tested this possibility in 15 of the MM samples that had shown strong (3+) MET overexpression by IHC without corresponding MET-GCNG/GA and in 5 samples with 3+ expression associated with MET-GCNG/GA (3 with intermediate-level and 2 with low-level, all EMMs). For this purpose, RNA was extracted from these specimens and analyzed by a well-established commercial method of targeted RNA-based next-generation sequencing (RNA-NGS; see Materials and Methods). The assay did not detect any MET exon 14 skipping mutations in the 5 MM cases with MET-GCNG/GA or in the 15 without MET-GCNG/GA, suggesting the presence of MET-overexpression mechanisms alternative to MET gene amplification or exon 14 skipping mutations in the latter tumors.

The RNA-NGS assay used in this study, in addition to MET exon 14 skipping, is designed to identify variants and fusions in the ALK, BRAF, EGFR, FGFR1-3, KRAS, MET, NRG1, NTRK1-3, RET and ROS1 genes. Thus, it is also pertinent to note that no variants or fusions affecting these oncogenic driver genes were identified in the 20 specimens (data not shown). These results are consistent with the paucity of oncogene activation reported in MM [7,8,9] and also indicate that MET-mutations or -fusions are unlikely to be the cause of MET overexpression in samples without MET-GCNG/GA. Moreover, they suggested that the cross talk of RTKs described in NSCLC, resulting in upregulation and activation of the MET receptor by mutant forms of other RTKs, such as those analyzed by our RNA-NGS panel [5,9], was unlikely to be involved in the analyzed MMs.

### 2.5. Correlation of MET Overexpression with Patient Outcome

**Figure 6:**
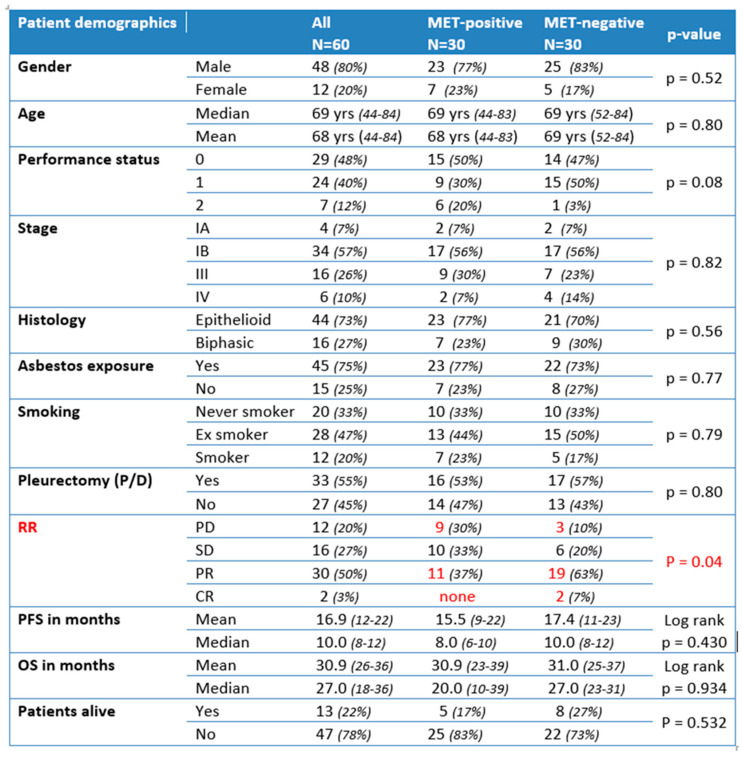
Correlation with clinicopathological data in patient-matched cohorts with (MET-positive) or without (MET-negative) MET upregulation (statistically significant differences highlighted in red). PD: progressive disease; SD: stable disease; PR: partial response; CR: complete response.

Having established that a significant fraction of MMs shows overexpression of MET receptor, though only partially correlating with MET-GCNG/GA, we were interested in assessing whether this overexpression had a prognostic impact and would predict a response to standard-of-care first-line chemotherapy in MM. For this purpose, we compared two groups of 30 consecutive, previously therapy-naïve patients with EMM or BMM who had been treated with first-line platinum-pemetrexed chemotherapy at our institution during the period 2015–2017. In each group, MET-expression status and MET-GCNG/GA had been assessed by IHC and FISH on diagnostic biopsies with >50% of tumor cell content, as described above. One patient group contained MMs displaying MET overexpression (immunoscore 2+/3+ = MET-positive) with or without concomitant FISH-detected MET-GCNG/GA, while in the other group the tumor tissue was without MET overexpression (immunoscore 1+/0 and no MET-GCNG/GA = MET-negative). As indicated in Figure 6, the two groups were matched concerning gender, age, performance status (PS), histological type, stage, asbestos exposure, smoking habit, and treatment (chemotherapy with/without following surgical resection by P/D) and thereby were not showing any significant difference in these parameters (Figure 6). Consistent with our previous observations, the MET-positive group showed MET overexpression by IHC only in epithelioid tumor cells, i.e., in EMMs and in the epithelioid but not sarcomatoid component of BMMs. Moreover, four of these 30 MET-overexpressing samples (13%) showed intermediate-level MET-GCNG/GA by FISH. The fact that this was a group that had been selected based on MET overexpression may explain why it showed a higher incidence of MET-GCNG/GA as compared to the larger unselected MM cohort described above.

**Figure 7:**
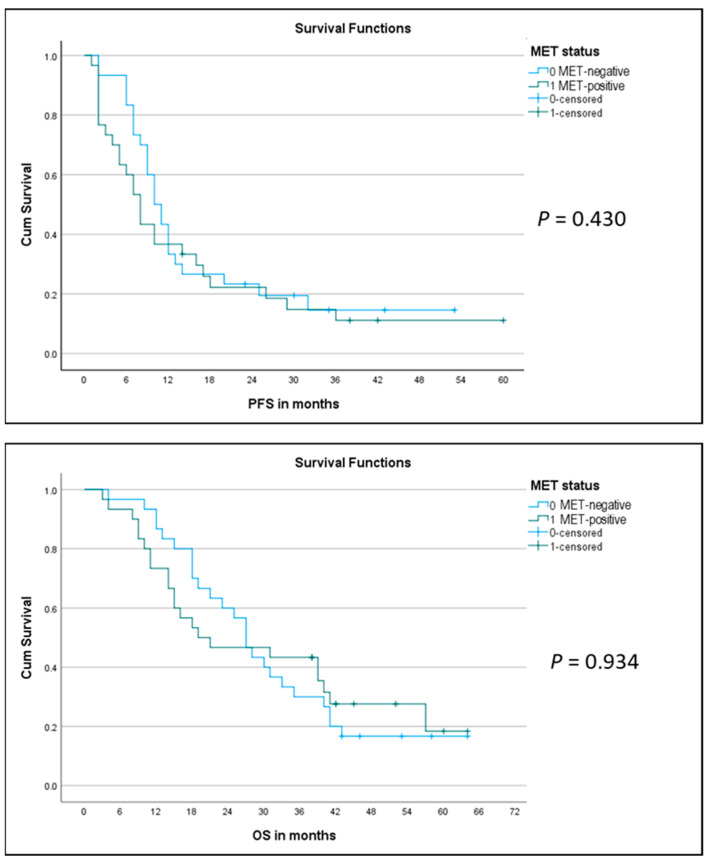
Kaplan–Meier curves for progression-free survival (PFS) (**top**) and for overall survival (OS) (**bottom**) in the two matched groups of MET-positive (green line) and MET-negative (blue line) MM patients.

The two matched groups were then compared with respect to outcome, evaluating the response rate (RR) to first-line platinum-pemetrexed chemotherapy (either neoadjuvant before P/D or for advanced disease), progression-free survival (PFS), overall survival (OS), and number of patients alive in the respective group. We noticed a significantly lower RR in the MET-positive group as compared to the MET-negative group, with fewer/no MET-positive patients showing partial/complete response and more patients with progressive disease as compared to MET-negative cases (responding MET-positive = 37% vs. responding MET-negative = 70%; *p* = 0.04) (Figure 6). As indicated on the Kaplan–Meier curve (Figure 7, top), there was no significant difference in PFS between the two groups (median PFS 8 months vs. 10 months; log-rank *p* = 0.430). Similarly, we did not observe a significant difference in OS between the two groups (MET-positive = median OS of 20 months; MET-negative = median OS of 27 months; log-rank *p* = 0.934) (Figure 6 and Figure 7, bottom). Furthermore, we observed fewer MET-positive patients alive after treatment as compared to MET-negative patients, but the difference was not statistically significant (17% MET-positive alive vs. 27% MET-negative alive; *p* = 0.532) (Figure 6).

In univariate analysis, female gender, stage IV, and lack of pleurectomy (P/D) showed a significantly negative impact on both PFS (Table 2) and OS (Table 3), whereas all the other variables, including MET status, did not exhibit any significant effect upon survival. A multivariate Cox regression analysis confirmed an independent negative impact upon PFS (Table 4) and OS (Table 5) for the female gender and for stage IV as compared to stages I–III, whereas an apparent positive impact on OS for pleurectomy did not reach statistical significance (Table 5). The other variables analyzed, including MET status, histological MM subtype, asbestos exposure, smoking status, PS, and age at diagnosis, did not exhibit a statistically significant independent effect on survival.

## 3. Discussion

In this study, based on a cohort of 155 consecutive MM patients, we used a well-established method of IHC [4,16,17] to identify cases having moderately (2+) or strongly (3+) increased MET protein expression in the tumor tissue. The latter was observed in 35% of patients. In contrast, we did not observe MET overexpression in RMPs, suggesting that upregulation of the receptor is related to a subset of MM, rather than simply cell proliferation. The MET overexpression appears significantly more common in EMM than in BMM and limited to the epithelioid MM cells, as we could detect it only in the epithelioid component of BMM, but not in sarcomatoid cells of BMMs or in SMM. More frequent overexpression of MET in the epithelioid subtype of MM has also been observed by others [13,14]. Interestingly, MET overexpression as assessed by IHC seems to poorly correlate with MET-GCNG/GA, given that low- or intermediate-level GCNG/GA were only observed in 27% of cases with a 3+ or 2+ MET IHC score. This poor correlation between protein expression and gene amplification suggests other mechanisms regulating MET expression and has also been observed by others in cases of NSCLC [4,5] and MM [14]. In NSCLC, the MET overexpression detected by IHC has usually been associated with MET-GCNG/GA, though MET exon 14 skipping mutations have also been described more recently as alternative and mutually exclusive mechanism of MET-receptor overexpression and activation that can be targeted by MET inhibitors [2,3,5]. Yet, the clinical studies have not completely clarified whether MET-receptor overexpression, MET-mutations, and MET-amplification might be interchangeable predictive biomarkers for MET-targeting therapy of NSCLC [4,5]. Data from clinical trials seem to suggest that MET-protein overexpression is less effective as a predictive biomarker in NSCLC than amplification or mutations of the MET gene, which could be explained not only by expression heterogeneity in tumor tissue and sample types, but also by the differences in IHC platforms, commercially available antibodies, and cutoffs for immunohistochemical positivity used in these clinical studies [4,5].

Likewise, previous immunohistochemical investigations of MET expression in MM have shown variable results attributable, at least in part, to the lack of standardized immunostaining and scoring procedures and to differences in types of specimens [13,14,15]. In the present study, we used both MM biopsies from chemotherapy-naïve patients and resection specimens taken after standard neoadjuvant platin-pemetrexed treatment for MM after having verified on patient-matched samples that the expression in the surgical material was not affected by the chemotherapy. Moreover, we applied a well-established method of immunostaining and scoring that we and others have previously utilized for assessing MET expression in NSCLC [16,17]. In contrast to NSCLC [4,16,17], we observed no significant heterogeneity of MET-protein expression in the MMs (i.e., cases with upregulated expression showed that in the vast majority of tumor cells). Also, differently from NSCLC, we did not find high-level MET-GCNG/GA by FISH in our MM cohort, consistent with the rarity of MET-amplification in previous studies performed by FISH or genomic profiling in pleural MM [7,8,14,15].

Yet, high-level MET-GCNG/GA might occur at low frequency also in MM, as a genomic analysis of 13 peritoneal MMs showed one case harboring focal high-level amplification of the MET oncogene, in addition to a structural rearrangement involving BAP1 and homozygous deletion of CDKN2A [12]. Furthermore, if we apply our FISH scoring criteria to previous reports, we notice that Bois et al. identified one case of high-level MET-GCNG/GA in a cohort of 149 analyzed pleural MMs [14], whereas Salvi et al. described at least 2 out of 106 MM cases (1.9%) in commercial tissue microarrays displaying high-level MET-GCNG/GA (MET/CEN7 ratio reportedly 4 and 6, respectively) [15]. These authors also reported 6 additional cases in their cohort that exhibited 6 to 10 MET copies in 60% to 80% of MM cells, which also fulfills our criteria for high-level MET-GCNG/GA. Overall, these results suggest that, although aberrations of BAP1, CDKN2A, and other tumor suppressor genes are the commonest genomic cancer-driving hits in MM [7,8,9], some rare cases of MM might also be driven by genomic dysregulation of MET signaling. In this respect, the level of MET-GCNG/GA in cancer cells may have importance for the intensity by which this event perturbs the MET-signaling pathway through protein overexpression and prolonged kinase activity [5]. Yet, what level (i.e., how many MET gene copies) should be reached in order to induce substantially increased MET-expression and ligand-independent sustained activation of MET signaling with clinically relevant oncogenic effect is unclear and may vary according to tumor type.

Most knowledge on the correlation between MET-gene copies and MET signaling in cancer patients derives from investigations in lung cancer. Indeed, in untreated NSCLC patients, low-/intermediate-level MET-GCNG/GA is often accompanied by co-mutations in other oncogenic drivers and is less sensitive to MET-signaling inhibition by MET-TKIs, as opposed to NSCLCs with high-level MET-GCNG/GA, which at baseline are less likely to harbor other detectable oncogenic drivers and are associated with significantly higher RR to MET-TKIs [4,5,16,23,24]. Accordingly, in the setting of EGFR-mutated NSCLC progressing on EGFR-TKIs, acquired resistance to EGFR-TKIs due to activation of the parallel bypass MET-signaling pathway is typically associated with high-level MET amplification [4,5]. These results suggest that high-level MET-GCNG/GA may act as an autonomous NSCLC-driver, which results in MET-dependence and the death of cancer cells, once the MET signaling to which they have become addicted is inhibited. Thus, high-level MET-GCNG/GA seems to be both a negative prognostic factor and a potential predictive biomarker for MET-TKIs [2,5,25]. In contrast, lower levels of MET-GCNG/GA may require a cooperative effect from other co-drivers in order to contribute to oncogenesis and it may therefore represent a weaker therapeutic target for NSCLC cells. However, whether this also applies to MM requires further studies exploring to what extent co-mutations may occur in MM with genomically deregulated MET and the possible response of these cases to MET inhibitors. In that respect, a limitation of our and previous studies is that they have not assessed by extensive genomic analysis the possible presence of co-mutations in the identified MM specimens harboring low-/intermediate-level MET-GCNG/GA. Accordingly, we do not know whether the pattern of genomic aberrations associated with various levels of MET-amplification in NSCLC is also present in MM.

A further drawback of the studies that have analyzed MET-GCNG/GA in MM by FISH is the lack of a standardized method for determining MET-amplification [13,14,15], which complicates their comparison. A similar problem has affected investigations of MET in NSCLC [4,5,25]. In the literature on different cancer types, MET-amplification has been defined as a ratio relative to chromosome 7 centromere (MET/CEN7 ratio) or as average MET-GCN/cell, which may theoretically include true gene amplification and high polysomy [4,5]. Although most FISH studies on MET alterations in cancer report either a MET/CEN7 ratio ≥2.0 or an average MET-GCN/cell of at least 4 in a tumor sample for categorizing it as MET-amplified, the parameters used and the cutoff to define “positivity” are not standardized and this may cause differences in the reported frequency of MET-amplification and its ability to be exploited as a potential therapeutic target [5]. Notably, some data suggest that an increased number of MET copies might be a better predictor of responses to MET-TKIs than the MET/CEN7 ratio [4,25].

Compared to other studies, we have classified the MM specimens according to a FISH-scoring method that we and others had successfully applied to NSCLC [16,17]. This approach considers both parameters used in other studies (MET/CEN7 ratio and average MET-GCN/cell) and uses reliable cutoffs for high-level amplification (MET/CEN7 ratio ≥2.0 and an average MET-GCN/cell ≥6.0). Moreover, it considers an additional parameter for high-level GCNG/GA, i.e., ≥10% of tumor cells with “clusters” of ≥15 MET signals and specific parameters for intermediate-level MET-GCNG/GA (≥50% of tumor cells with ≥5 MET signals) and for low-level MET-GCNG/GA (≥40% of tumor cells with ≥4 MET signals). The special emphasis on average GCN and percentages of tumor cells with ≥4, ≥5, and ≥15 MET-GCN/cell as well as the fact that the method is based on the analysis of neighboring tumor cell nuclei from five random areas of homogenous distribution of MET signals [16,17], enables to classify the samples, including those with heterogeneous MET-GCNG/GA, at different MET-amplification levels. Importantly, the approach avoids excluding the samples, in which MET-GCNG/GA may occur with co-amplification of the centromeric 7 region and thereby results in a paradoxical “negative” MET/CEN7 ratio <2.0 [16,17,25]. Given its robustness, this FISH scoring method has been adopted in clinical trials [25,26]. Recently, Overbeck et al. have added a top-level category of MET-GCNG/GA in NSCLC, corresponding to an average MET-GCN/cell ≥10, which was observed in a subset of poorly differentiated adenocarcinomas with unfavorable prognosis [25]. However, having not observed any high-level MET-GCNG/GA in our study, which began before the work of Overbeck et al. was published, we did not include top-level MET-GCNG/GA in the classification of our MM cohort.

MET splice mutations result in MET overexpression and are reportedly mutually exclusive with MET-amplification in NSCLC [2,3,4,5]. However, we did not detect any MET exon 14 skipping mutations in MM cases showing MET overexpression without concomitant MET-GCNG/GA. These cases did not harbor MET-fusions either. These observations suggest the possibility that transcriptional/posttranscriptional/epigenetic mechanisms rather than MET gene amplification, exon 14 skipping mutations at splice sites or fusions could have caused the receptor overexpression in these tumors. Further studies are necessary to address the causes of MET overexpression in MM.

Importantly, despite the limitations of the study, including also the small size (affecting the statistical power) of the two matched cohorts of MET-positive and MET-negative MM patients, our data indicate that MET-protein overexpression was associated with lower RR to standard platinum-pemetrexed chemotherapy for MM, while no significant difference in median PFS and median OS after standard chemotherapy was observed between the two groups. Moreover, a multivariate regression analysis revealed an independent significant impact on survival for female gender (possibly due to the low number of females in our cohorts) and MM stage, but not for the MET status. We noticed also an apparent positive impact on survival for P/D, which, though, did not reach statistical significance, possibly due to the low number of pleurectomized patients in the analysis.

Our results imply that MET overexpression occurs in a substantial fraction of predominantly epithelioid MMs, and that despite correlating poorly with MET-amplification status, it may negatively impact the RR of MM patients receiving standard chemotherapy. This may be due to MET being a driver of tumor cell proliferation/survival/invasiveness [1,2], and to some extent causing insensitivity to chemotherapy. Larger cohorts are needed to reach conclusions regarding the impact of MET alterations on the survival of MM patients. In this regard, our results may suggest a possibility of treating MET-positive MM by MET inhibitors, as currently evaluated in other malignancies, e.g., in NSCLC, but would need validation in another and larger cohort beforehand. However, in NSCLC there seems to be a MET gene dose effect (the higher MET-GCN/cell, the higher are chances of response to MET-TKIs), causing uncertainty regarding which levels of MET-receptor overexpression and MET-gene amplification are actionable targets in NSCLC [4,5,25]. Therefore, the extent of these MET alterations in MM and the predictive significance of MET-IHC and -FISH for possible MET-targeted therapy of MM also remain to be elucidated.

## 4. Materials and Methods

### 4.1. Tissue Samples

The study examined a cohort of 155 consecutive MM cases treated at our institution between 2015 and 2017 with demographic and pathological characteristics, as specified in Table 1. The cohort comprised 110 diagnostic biopsies of treatment-naïve MM (97 pleural and 13 peritoneal) and 45 extended pleurectomies/decortications (P/Ds) performed at our institution after 3 courses of neoadjuvant cisplatin-pemetrexed. All the included MM samples from the cohort were formalin-fixed, paraffin-embedded (FFPE), had a tumor cell content [(number tumor cell nuclei: total number of cell nuclei) × 100] of >50%, and had been diagnosed according to the criteria defined by the World Health Organization (WHO) and the International Mesothelioma Interest Group [27,28,29]. The specimens included 77 epithelioid MMs (EMMs: 68 pleural, 9 peritoneal), 68 biphasic MMs (BMMs: 64 pleural, 4 peritoneal), and 10 sarcomatoid MMs (SMMs: all pleural). Thirty-three RMPs identified in FFPE surgical samples from patients operated for non-malignant pulmonary/mediastinal disease were used as non-neoplastic controls for the three types of MM (Table 1): pleura on pulmonary wedge-resection after pneumothorax due to ruptured cyst/bulla (n = 24); lung explant/pneumonectomy for end-stage sarcoidosis or chronic allergic alveolitis (n = 3); pleura-covered resection of thymus for thymus hyperplasia (n = 1) or of aorta for dissection (n = 2); pericardial cyst (n = 3). In all these samples, we identified variable pleural or pericardial chronic inflammation associated with mesothelial hyperplasia and fibrosis.

### 4.2. Immunohistochemistry

The immunostaining for membranous and cytoplasmic expression of MET-receptor was performed as previously described [17]. Briefly, 2.5-μm-thick FFPE tissue sections from each sample were stained using a Roche-Ventana BenchMark ULTRA automated slide immunostainer (Ventana Medical Systems Inc.; Roche Diagnostics A/S, Hvidovre, Denmark), Ultra Cell Conditioning solution (CC1) pretreatment for 8 min at 95 °C, four CC1 treatments (20, 36, 52, and 64 min), and incubation with the pre-diluted CONFIRM anti-MET (clone ID, SP44) rabbit monoclonal antibody (mAb) (Ventana Medical Systems, Inc.; Roche Diagnostics A/S, Hvidovre, Denmark) for 16 min. The immunostaining for the mesothelial marker calretinin was carried out on specific tissue sections to further visualize malignant or hyperplastic mesothelial cells using CC1 pretreatment for 64 min at 97 °C followed by incubation for 32 min at 36 °C with anti-calretinin rabbit mAb (clone ID, SP65; Ventana Medical Systems Inc.; Roche Diagnostics A/S, Hvidovre, Denmark) at 1:400 dilution, as previously reported [20]. The immune reactions were visualized using ultraView DAB Detection Kit (Ventana Medical Systems, Inc.; Roche Diagnostics A/S, Hvidovre, Denmark) and hematoxylin counterstaining (Ventana Medical Systems, Inc.; Roche Diagnostics A/S, Hvidovre, Denmark) following the manufacturer’s recommendations.

MET-protein expression was scored in a blinded manner (without knowing the FISH results) by one observer (E.S.-R.), assessing staining intensity (negative, weak, moderate or strong) and the percentage of stained cells, thereby defining four diagnostic “immunoscores”, i.e., 3+ (strong intensity in ≥50% of cells); 2+ (moderate intensity in ≥50% of cells); 1+ (weak intensity in ≥50% of tumor cells); 0 (no staining or <50% of tumor cells stained), and considering 2+/3+ as indicative of MET upregulation (“MET-positive”) as opposed to no upregulation (“MET-negative”), as previously described [16,17]. Endothelial cells or bronchial/alveolar epithelial cells present in the tissue sections were used as internal controls, since they can display weak and weak-moderate intensity of MET expression, respectively, as reported [16,17] (example in Supplementary Figure S1). Image acquisition was obtained by digital scanning of the slides with a Nano Zoomer S210 slide scanner (Hamamatsu, Ballerup, Denmark) and the digital slide viewing software Sectra Workstation IDS7 (Sectra AB, Linköping, Sweden).

Before staining the cohort of MM cases, we performed a smaller pilot investigation comparing the MET immunostaining of 30 independent FFPE diagnostic biopsies of MM with their corresponding patient-matched P/D specimens, in order to assess whether the neoadjuvant chemotherapy would have any effect on MET expression in P/D specimens as compared to the corresponding chemotherapy-naïve diagnostic biopsies. For this purpose, we selected from samples utilized in previous studies [20,21,22] 10 diagnostic biopsies displaying MET-negative expression (0/1+, as defined above), 10 displaying a MET immunoscore of 2+, and 10 displaying an immunoscore of 3+, and then compared these results with the immunostainings in their corresponding patient-matched P/D specimens.

### 4.3. Fluorescence In Situ Hybridization

FISH was performed with the Zyto-Light SPEC MET/CEN7 dual-color probe (Zytovision GmbH, AH diagnostics A/S, Tilst, Denmark) that detects the MET gene and the centromeric portion of the MET-harboring chromosome 7, as we previously described [17]. Briefly, slides were scanned using a ×63 objective and appropriate filter sets (automated upright Leica DM4 B fluorescent microscope; Leica Microsystems, Brønshøj, Denmark), using normal fibroblasts, leukocytes, endothelial cells or non-neoplastic lung tissue as internal controls and individually analyzing 100 tumor cell nuclei (20 neighboring tumor cell nuclei from five random areas of homogenous distribution of MET signals) with the ×100 objective, counting MET (green) and CEN7 (orange) signals. Representative images were acquired using the 19 mm sCMOS Leica DFC9000 camera incorporated with the microscope after identification of representative areas with the Leica LAS X Navigator Software (Leica Microsystems, Brønshøj, Denmark). FISH was assessed by two readers (E.S.-R. and a trained and experienced laboratory technician) without knowing the IHC data. The samples were classified into the following four groups of MET-amplification status [16,17]: (A) High-level MET-GCNG/GA = MET/CEN7 ratio ≥2.0 or an average MET-GCN/cell ≥6.0 or ≥10% of tumor cells with ≥15 MET signals (“clusters”); (B) Intermediate-level MET-GCNG/GA = ≥50% of tumor cells with ≥5 MET signals; (C) Low-level MET-GCNG/GA = ≥40% of tumor cells with ≥4 MET signals; (D) No MET-GCNG/GA = none of above criteria fulfilled.

### 4.4. RNA Next-Generation Sequencing of MET Exon 14 Skipping Mutations

For analysis of possible mutations causing an aberrantly spliced transcript of MET leading to exon 14 skipping and reduced degradation of the MET-receptor [3,4,5], total RNA was extracted from the MM specimens showing MET overexpression (3+) using a Maxwell RSC RNA FFPE kit (Promega, Madison, WI, USA). RNA-NGS was performed using the Archer FusionPlex^®^ Lung kit according to the manufacturer’s instructions (ArcherDX, Inc., Boulder, CO, USA). In addition to MET exon 14 skipping mutations, this method allows the detection of variants and fusions involving the ALK, BRAF, EGFR, FGFR1-3, KRAS, MET, NRG1, NTRK1-3, RET and ROS1 genes.

### 4.5. Correlation of MET-Amplification and Expression with Treatment Outcome

To assess the prognostic impact or predictive value for response to chemotherapy of MET-amplification and/or overexpression we compared two MM patient groups with and without MET aberrations treated during 2015–2017. For this purpose, MET-expression status and MET-GCNG/GA were evaluated as described above on FFPE diagnostic tissue biopsies obtained from 60 consecutive, previously untreated patients with EMM or BMM. Tumor samples from 30 of these 60 patients displayed MET overexpression by IHC (MET-positive = immunoscores 2+/3+) with or without concomitant MET-GCNG/GA detected by FISH. MM samples from the other 30 patients had an immunoscore of 1+/0 (MET-negative) and no MET-GCNG/GA. The two groups of patients were matched concerning gender, age, performance status, histologic subtypes, stage, asbestos exposure, smoking habit, and treatment (chemotherapy alone or chemotherapy plus P/D) and were compared with respect to outcome, such as RR to chemotherapy, PFS, OS, and number of patients alive in the respective group.

### 4.6. Statistical Analysis

For immunohistochemical data, the unpaired Student’s *t*-test (two-tailed) was used to detect significant differences in expression between two groups. Significant differences in the MET expression in three or more groups overall were detected using one-way ANOVA. *p* values adjusted for mass significance were obtained using Tukey–Kramer post-tests. For the analysis of IHC results, the distributions of H-scores in three groups were compared using nonparametric Kruskal–Wallis ANOVA and the Dunn post-test. For the correlation with outcome and the analysis of alive patients in the two groups of MET-positive and MET-negative patients a Chi-squared test was performed. OS was defined as the time from diagnosis to the time of death from any cause or last follow-up. PFS was defined as the time from diagnosis to a documented progression or death from any cause. For patients without any progression at the time of analysis, the date of last follow-up was considered right-censored. OS and PFS analysis were performed using the Kaplan–Meier method and survival curves were tested for differences by using the log-rank test. The possible independent effect upon survival of the variables considered in the study was assessed by multivariate Cox regression analysis. A *p*-value < 0.05 was considered statistically significant. Statistical analyses were performed using IBM Statistics SPSS software (IBM Corp., IBM SPSS Statistics for Windows, Version 25.0. Armonk, NY, USA).

## Figures and Tables

**Table 1:** Demographic and pathological features of patients with MM or RMP.

Characteristics	MPM P/D (n = 45)	MM Biopsy (n = 110)	RMP (n = 33)
**Gender**			
Male (%)	39 (87)	88 (80)	25 (76)
Female (%)	6 (13)	22 (20)	8 (24)
**Age, mean (range)**	68 (40–74)	70 (40–86)	39 (17–79)
**Smoking history**			
Ex/current smoker (%)	29 (64)	83 (75)	20 (60)
Non-smoker (%)	16 (36)	27 (25)	13 (40)
**Asbestos exposure**			
Yes (%)	34 (76)	77 (70)	0
No (%)	11 (24)	33 (30)	33 (100)
**Chemotherapy (cisplatin + pemetrexed) (%)**	45 (100)	0	0
**Histological type**			
Epithelioid (%)	20 (44)	57 (52)	
Biphasic (%)	25 (56)	43 (39)	
Sarcomatoid (%)	0	10 (9)	
**Site**			
Pleura (%)	45 (100)	97 (88)	33 (100)
Peritoneum (%)		13 (12)	

MM biopsy: diagnostic biopsy from malignant mesothelioma; MPM P/D: malignant pleural mesothelioma removed by pleurectomy/decortication; RMP: reactive mesothelial proliferation.

**Table 2:** Univariate analysis of PFS.

	Hazard Ratio (HR)	95% CI (HR)	*p*-Value
MET status	1.240	0.714–2.153	0.446
Gender	0.419	0.211–0.833	* 0.013
Histologic type	1.012	0.552–1.854	0.970
Asbestos exposure	0.808	0.429–1.523	0.510
Smoking status	0.946	0.532–1.682	0.851
Stage	3.336	1.366–8.147	* 0.008
Performance status	1.387	0.590–3.259	0.454
Pleurectomy (P/D)	0.508	0.290–0.892	* 0.018
Age	1.005	0.970–1.041	0.774

P/D: pleurectomy/decortication. Significant *p* values are marked by *.

**Table 3:** Univariate analysis of OS.

	Hazard Ratio (HR)	95% CI (HR)	*p*-Value
MET status	0.976	0.549–1.735	0.935
Gender	0.407	0.205–0.809	* 0.010
Histologic type	0.931	0.489–1.773	0.828
Asbestos exposure	0.962	0.499–1.855	0.909
Smoking status	0.970	0.527–1.783	0.921
Stage	2.659	1.108–6.382	* 0.029
Performance status	2.174	0.916–5.162	0.078
Pleurectomy (P/D)	0.385	0.213–0.694	* 0.002
Age	1.024	0.987–1.061	0.206

P/D: pleurectomy/decortication. Significant *p* values are marked by *.

**Table 4:** Multivariate analysis of PFS.

	Hazard Ratio (HR)	95% CI (HR)	*p*-Value
MET status	1.356	0.763–2.413	0.300
Gender	0.299	0.128–0.697	* 0.005
Histologic type	1.278	0.645–2.531	0.483
Asbestos exposure	1.038	0.495–2.176	0.921
Smoking status	1.460	0.741–2.878	0.274
Stage	3.911	1.364–11.215	* 0.011
Performance status	2.110	0.770–5.779	0.146
Pleurectomy (P/D)	0.576	0.286–1.160	0.123
Age	0.976	0.938–1.015	0.226

P/D: pleurectomy/decortication. Significant *p* values are marked by *.

**Table 5:** Multivariate analysis of OS.

	Hazard Ratio (HR)	95% CI (HR)	*p*-Value
MET status	1.051	0.563–1.962	0.877
Gender	0.223	0.085–0.586	* 0.002
Histologic type	1.439	0.688–3.009	0.334
Asbestos exposure	1.923	0.825–4.478	0.130
Smoking status	1.551	0.723–3.325	0.260
Stage	3.319	1.045–9.425	* 0.041
Performance status	2.110	0.770–5.779	0.146
Pleurectomy (P/D)	0.471	0.217–1.024	0.057
Age	0.980	0.937–1.025	0.375

P/D: pleurectomy/decortication. Significant *p* values are marked by *.

## Data Availability

All datasets generated during and/or analyzed during the current study are available from the corresponding author on reasonable request within the legal use of confidential data.

## Supplementary Materials

The following materials are available online at https://www.mdpi.com/article/10.3390/ijms222312868/s1.
