# Supplementary figures and images for: Correlation of MET-Receptor Overexpression with MET Gene Amplification and Patient Outcome in Malignant Mesothelioma
Source: Int J Mol Sci. 2021 Nov 28;22(23):12868. doi: 10.3390/ijms222312868 (PMC8657838; doi:10.3390/ijms222312868)

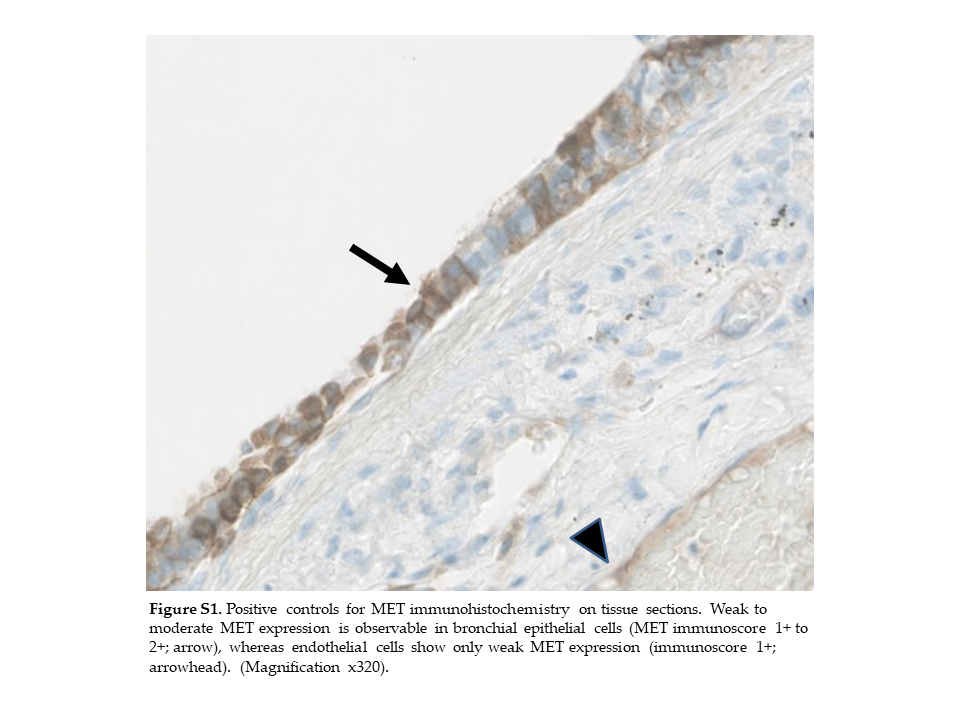

Supplement: Supplementary file 1 [file ijms-22-12868-s001.zip › Figure S1.tif]

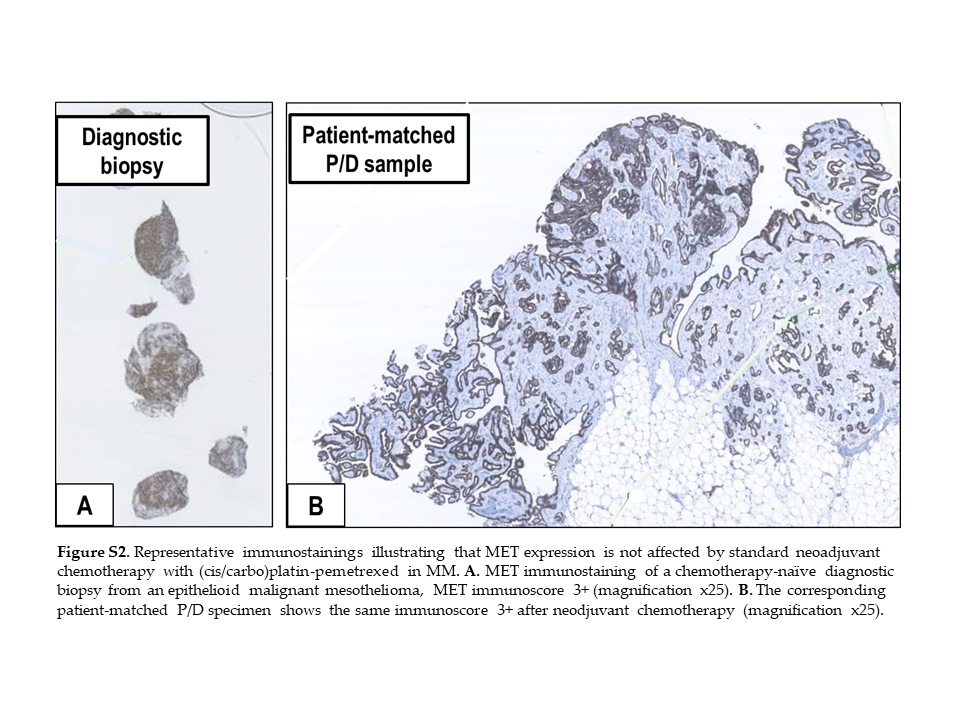

Supplement: Supplementary file 1 [file ijms-22-12868-s001.zip › Figure S2.tif]

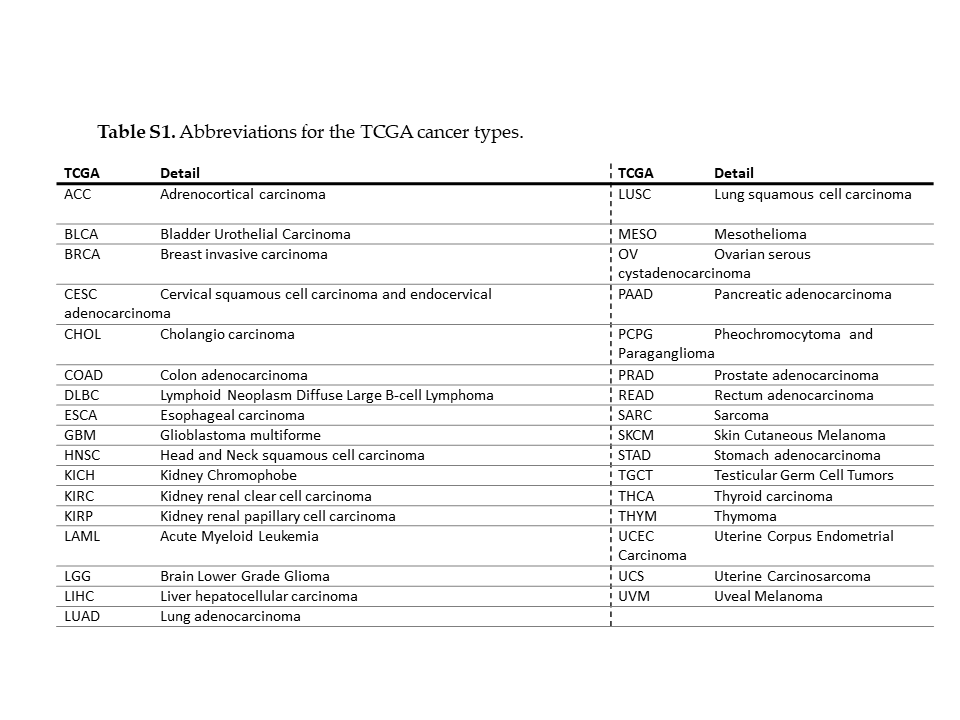

Supplement: Supplementary file 1 [file ijms-22-12868-s001.zip › Table S1.tif]
